# Response to vitamin d replacement in overweight and normal weight children with vitamin D deficiency

**DOI:** 10.1186/1687-9856-2015-S1-P76

**Published:** 2015-04-28

**Authors:** Haejung Kim, In Hyuk Chung, Eun-Gyong Yoo

**Affiliations:** 1 Department of Pediatrics, CHA University, Sungnam, Korea; 2 Department of Pediatrics, National Health Corporation, Ilsan Hospital, Ilsan, Korea

## Aims

Obesity is a risk factor for vitamin D deficiency (VDD), because the lipid soluble vitamin D can be sequestered in adipose tissue. Although it was suggested that higher dose of vitamin D might be required to treat VDD in obese individuals, little is known about treatment responses in overweight children. We investigated the response to vitamin D replacement in normal weight and overweight children.

## Methods

This is a prospective study including 66 Korean children between 8 and15 years of age diagnosed with VDD between Dec 2013 and Feb 2014. VDD was defined as serum 25OHD < 20 ng/mL, and vitamin D sufficiency as ≥30 ng/mL. Overweight was defined as body mass index (BMI) ≥85^th^ percentile (n = 25), and normal weight as BMI between 5^th^ and 84^th^ percentile (n = 41). All participants received vitamin D_3_ supplementation (2000 IU/d) for 8 weeks. The level of serum 25OHD, PTH, and biochemical parameters were measured before and after treatment.

## Results

The mean age was 9.9 ± 1.4 years in normal weight children and 10.0 ± 2.1 years in overweight children (p=ns). Baseline serum 25OHD level was lower in normal weight children (13.2 ± 3.2 ng/mL) than in overweight children (14.2 ± 2.1 ng/mL, p=0.011). Baseline PTH level was 32.3 ± 9.5 and 39.5 ± 18.0 pg/mL in normal weight and overweight children, respectively (p=0.027). After 8 weeks of treatment, 28 (68.3%) normal weight children and 10 (40%) overweight children achieved vitamin D sufficiency (p=0.023). The mean serum 25OHD level was 33.7 and 28.6 ng/mL in normal weight and overweight children, respectively (p=0.496). The increase of 25OHD levels after treatment was significantly higher in normal weight children than in overweight children (20.6 ± 7.2 vs. 14.4 ± 7.9 ng/mL, p=0.002). However, the decrease in PTH levels seemed to be slightly larger in overweight children compared to normal weight children (-3.2 ± 20.8 vs. -1.1 ± 11.1 pg/mL, p=0.05). In multiple regression analysis, overweight was significantly related to the 25OHD increase after vitamin D replacement (β=0.323, p=0.01).

## Conclusion

The response to vitamin D replacement can be influenced by adiposity, and overweight children require larger doses of vitamin D to achieve vitamin D sufficiency.

